# Transformation of Undifferentiated Thy-1^lo^ B220^+^ Thymic Lymphoid Cells by the Abelson Murine Leukemia Virus

**DOI:** 10.1155/2000/94158

**Published:** 2000

**Authors:** Eric Y. Chen, Bradley J. Swanson, Steven S. Clark

**Affiliations:** 1 Department of Human Oncology and the University of Wisconsin Comprehensive Cancer Center University of Wisconsin Madison WI USA; 2 Department of Human Oncology University of Wisconsin K4/432 Clinical Science Center 600 Highland Ave. Madison WI 53792 USA

**Keywords:** Abelson Murine Leukemia Virus, A-MuLV, lymphocyte progenitor, Ig genes, T cell receptor genes, thymic lymphoma

## Abstract

Intrathymic injection of the Abelson murine leukemia virus (A-MuLV) results in transformation of immature T and B lymphoid cells. In this report we demonstrate that the concentration of A-MuLV injected into murine thymi influences the selection of the transformation target. Thus, concentrated A-MuLV gives rise to Thy-1^+^ B220^-^ thymomas. In contrast, dilute virus induces B220^+^ thymomas that also express low levels of Thy- 1 (Thy-1^lo^), a phenotype that is similar to marrow-derived progenitor B-lymphoid cells (pro-B cells) that are highly susceptible to A-MuLV transformation *in* *vitro*. However, rare B220^+^ lymphoid cells isolated from normal adult thymi were not transformed by A-MuLV *in* *vitro*, while B220^+^ cells isolated from bone marrow were highly susceptible to transformation by A-MuLV. The Thy-1^lo^ B220^+^population in the primary thymomas had not rearranged TCRγ, TCRβ, or Igκ genes, but contained subpopulations that assembled *Ig* DJ_H_ or VDJ_H_ genes and were therefore similar to transformed pro- and pre-B cells obtained from A-MuLV infected fetal liver and adult bone marrow, respectively. However, unlike A-MuLV-transformed pro- and pre-B cells, many (40– 70%) of the Thy-1^lo^ B220^+^ transformed thymoma cells had not rearranged *Igh* genes, and therefore appear to represent undifferentiated lymphoid cells. We conclude that A-MuLV may transform an undifferentiated lymphoid target in the thymus.


*Developmental Immunology*, 2000, Vol. 8(1), pp. 1-18
Reprints available directly from the publisher
Photocopying permitted by license only

© 2000 OPA (Overseas Publishers Association)
N.V. Published by license under the
Harwood Academic Publishers imprint,
part of The Gordon and Breach Publishing Group.
Printed in Malaysia

### Transformation of Undifferentiated Thy-$1^{lo}$ B220$^{+}$ Thymic Lymphoid Cells by the Abelson Murine Leukemia Virus

ERIC Y. CHEN, BRADLEY J. SWANSON and STEVEN S. CLARK*

*From the Department of Human Oncology and the University of Wisconsin Comprehensive Cancer Center; M.D./Ph.D. Integrated Degree Program, Human Cancer Biology Training Program and Program in Cell and Molecular Biology, University of Wisconsin, Madison, WI*

Intrathymic injection of the Abelson murine leukemia virus (A-MuLV) results in transformation of immature T and B lymphoid cells. In this report we demonstrate that the concentration of A-MuLV injected into murine thymi influences the selection of the transformation target. Thus, concentrated A-MuLV gives rise to Thy-$1^{+}$ B220$^{-}$ thymomas. In contrast, dilute virus induces B220$^{+}$ thymomas that also express low levels of Thy- 1 (Thy-$1^{lo}$), a phenotype that is similar to marrow-derived progenitor B-lymphoid cells (pro-B cells) that are highly susceptible to A-MuLV transformation *in vitro*. However, rare B220$^{+}$ lymphoid cells isolated from normal adult thymi were not transformed by A-MuLV *in vitro*, while B220$^{+}$ cells isolated from bone marrow were highly susceptible to transformation by A-MuLV. The Thy-$1^{lo}$ B220$^{+}$population in the primary thymomas had not rearranged *TCRγ*, *TCRβ*, or *Igκ* genes, but contained subpopulations that assembled *Ig* $DJ_H$ or $VDJ_H$ genes and were therefore similar to transformed pro- and pre-B cells obtained from A-MuLV infected fetal liver and adult bone marrow, respectively. However, unlike A-MuLV-transformed pro- and pre-B cells, many (40–70%) of the Thy-$1^{lo}$ B220$^{+}$ transformed thymoma cells had not rearranged *Igh* genes, and therefore appear to represent undifferentiated lymphoid cells. We conclude that A-MuLV may transform an undifferentiated lymphoid target in the thymus.

*Keywords:* Abelson Murine Leukemia Virus, A-MuLV, lymphocyte progenitor, Ig genes, T cell receptor genes, thymic lymphoma

Supported by NIH guants CA52142, CA09471, and CA14520, by the Maxfield Foundation and by a genecous gift to the UW Comprchensive Cancer Center by Roger and Jeanne DeMeritt

### INTRODUCTION

The ability of the v-*abl* oncogene of the Abelson murine leukemia virus (A-MuLV) to immortalize immature lymphoid cells from bone marrow and fetal liver has been useful for identifying and characterizing immature stages of B-cell development (Rosenberg and Witte, 1988; Alt et al., 1984). Depending on the extent of *Igh* rearrangement, most transformed cell lines derived from A-MuLV infected bone marrow or fetal liver can be divided into one of three categories. Cell lines in the most common category

* Corresponding author: Dr. Steven S. Clark, Department of Human Oncology, University of Wisconsin, K4/432 Clinical Science Center, 600 Highland Ave., Madison, WI 53792. Phone: 608/263–9137; EMail: ssclark@facstaff.wisc.edu represent transformed pre-B cells that exhibit complete variable region formation on at least one allele. However, because of the imprecision of $V_HDJ_H$ recombination, these transformants may or may not express cytoplasmic mu chains (C$\mu$) (Alt et al., 1984). Even if a functional *Igh* variable region is formed and C$\mu$ is expressed, these pre-B cell transformants are usually arrested at this stage of B-cell differentiation by v-*abl* suppression of *RAG* gene expression and/or inhibition of NF-$\kappa$B/*rel*-mediated $\kappa$ intron enhancer activity (Chen et al., 1994; Klug et al., 1994).

A second marrow-derived A-MuLV-transformed cell type is arrested at the $DJ_H$ stage of maturation. These cells fail to rearrange $V_H$ to $DJ_H$ and are referred to as DJ-fixed (DJ-f) (Ramakrishnan and Rosenberg, 1988; Wang and Rosenberg, 1993). A-MuLV transformed cell lines derived from fetal liver also have rearranged only $DJ_H$ segments, but unlike the DJ-f population obtained from marrow, are not blocked at this stage of development and continue assembling *Igh* genes so that ≤10% of the cells express C$\mu$. This third type of A-MuLV transformant is referred to as DJ-rearranging (DJ-r) (Ramakrishnan and Rosenberg, 1988).

Thymic lymphocytes can also be transformed by A-MuLV (Clark et al., 1993; Cook, 1982; Cook, 1985; Cook and Balaton, 1987; Holland et al., 1991; Kimoto et al., 1989; Risser et al., 1985; Scott et al., 1986; D'Andrea et al., 1987), and these can be divided into three categories based on their level of Thy-1 expression (Clark et al., 1993; Cook, 1982; Cook, 1985; Cook and Balaton, 1987; Scott et al., 1986). Transformed thymocytes that express high levels of Thy-1 (Thy-$1^{hi}$) are also B220$^-$ and have rearranged their TCR$\gamma$ and $\beta$ genes. These represent early-to-intermediate stages of $\gamma/\delta$ or $\alpha/\beta$ T-cell development (Clark et al., 1993; Cook, 1982; Cook, 1985; Cook and Balaton, 1987; Holland et al., 1991; Risser et al., 1985; Scott et al., 1986). Other A-MuLV transformed thymic lymphocytes do not express Thy-1 (Cook, 1982; Cook, 1985; Cook and Balaton, 1987; Kimoto et al., 1989; Risser et al., 1985; Scott et al., 1986; D'Andrea et al., 1987), and when examined, express B220 (Kimoto et al., 1989; Risser et al., 1985; Scott et al., 1986). These have extensively rearranged *Igh* genes and often have rearranged *Ig*$\kappa$ and *TCR*$\gamma$ genes, but maintain unrearranged TCR$\beta$ genes (Cook and Balaton, 1987; Kimoto et al., 1989; Scott et al., 1986; D'Andrea et al., 1987). Therefore, this A-MuLV-transformed thymocyte population consists of pre-B cells, similar to the common marrow-derived A-MuLV transformant. Thus, rare B-progenitor cells that can be transformed by A-MuLV appear to exist in the thymi of adult mice. The origin of these cells and their relationship to marrow-derived B-progenitor cells are not understood at this time.

Also of interest are the relatively rare A-MuLV-induced thymoma-derived cell lines reported to express low levels of Thy-1 (Thy-$1^{lo}$)(Cook, 1982; Cook, 1985; Cook and Balaton, 1987; Holland et al., 1991) because in murine hematolymphoid populations, Thy-$1^{lo}$ often characterizes progenitor and stem cells (Kondo et al., 1997; Lian et al., 1997; McKenna and Morrissey 1998; Morrison and Weissman 1994; Müller-Sieburg et al., 1986; Soloffet al. 1997). In addition, Thy-$1^{lo}$ is also found expressed on early thymic progenitors (Antica et al. 1994; Carlyle et al. 1997; Hattori et al. 1994; Kawamoto et al. 1999; Wu et al. 1991). Unlike the other categories of A-MuLV-transformed thymoma cells that clearly represent distinct stages of T or B-cell development, the few A-MuLV-transformed Thy-$1^{lo}$ cell lines that have been examined express an ambiguous phenotype that cannot unequivocally be assigned to a particular stage of T or B lymphocyte development (Cook, 1985; 1987; Cook and Balaton, 1987; Risser et al., 1985; D'Andrea et al., 1987; Holland et al., 1991). These thymoma cells may be analogous to cases of acute human leukemia in which malignant lymphoblasts with an ambiguous phenotype are thought to arise from the transformation of a transitory lymphoid progenitor poised at the juncture of commitment to either the T or B-lymphoid lineage (Paietta, 1992; Greaves, 1986). Since progenitor populations capable of giving rise to both T and B-cells can be found in the thymi of adult and fetal mice (Akashi et al. 1998; Hattori et al. 1994; Matsuzaki et al., 1993; Peault et al. 1994; Wu et al., 1991), it is feasible that primitive lymphoid progenitor cells not fully committed to either lymphoid lineage may be targets for A-MuLV transformation in the thymus.

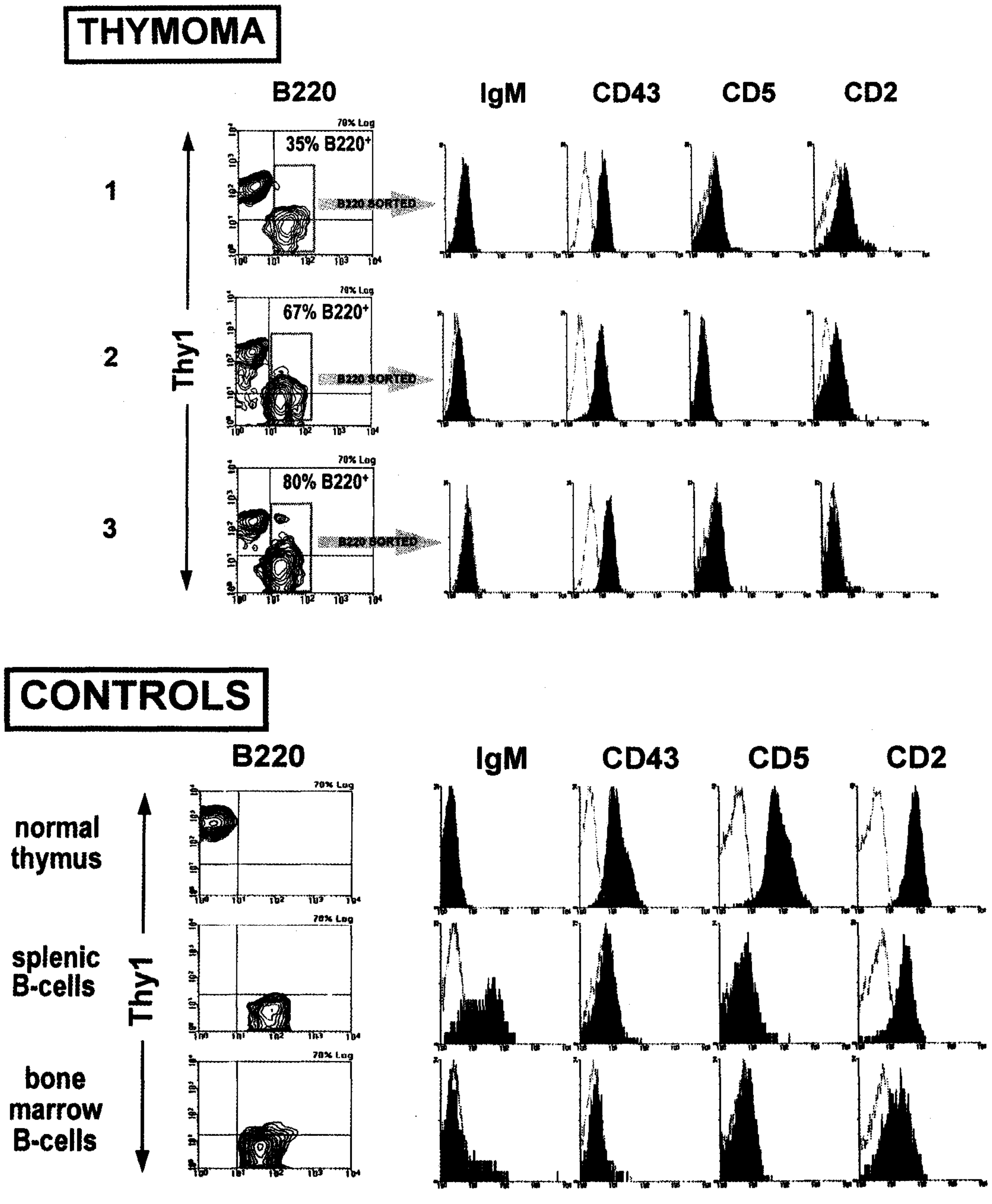

FIGURE 1 Coexpression of B220 and Thy-$1^{lo}$ cells on immature lymphocytes in primary thymomas induced by A-MuLV. Three representative thymomas in which 35%, 67% and 80% of the cells were $B220^+$ were analyzed for coexpression of B220 and Thy-1 by two color flow cytometry as described in the Materials and Methods. The data show that the $B220^+$ cells expressed low levels of Thy-1, and that $B220^-$ cells were Thy-$1^{hi}$. This is representative of 22 thymic tumors analyzed. $B220^+$ cells were also sorted to ≥95% purity over the anti-B220 magnetic column then stained with (solid histograms) FITC-R6–60.2 (anti-IgM), FITC-S7 (anti-CD43), PE-53–7.3 (anti-CD5), PE-RM2–5 (anti-CD2), or PE-M1/69 (anti-HSA) (data not shown) and analyzed by flow cytofluorometry. Isotype-matched irrelevant antibodies were used to establish background fluorescence (open histograms). These data are representative of nine Thy-$1^{lo}$ $B220^+$ tumor populations that were examined. Staining profiles for normal thymus and electronically gated apleen and bone marrow $B220^+$ cells are included for control.

While comparing thymomas induced by intrathymic injection of different retroviruses, we noticed that distinct Thy-$1^{lo}$ and Thy-$1^{hi}$ populations often co-existed in the same primary tumor that was induced with either the v-*abl* or *BCR-ABL* oncogenes. In contrast, we only observed Thy-$1^{hi}$ cells in tumors induced with the Moloney murine leukemia virus (Clark et al., 1993). The Thy-$1^{lo}$ tumor cells could represent the *in vivo* counterpart of the rare Thy-$1^{lo}$ A-MuLV transformed thymoma derived cell lines that are of an undetermined stage of lymphocyte development (Cook, 1985; Cook and Balaton, 1987; Holland et al., 1991). Since the Thy-$1^{lo}$ category of A-MuLV transformed thymomas may be relevant to early lymphocyte development (Clark et al., 1993; Cook, 1985; Cook and Balaton, 1987; Holland et al., 1991), it is important to better characterize these tumor cells and the normal thymocyte population that gives rise to them. Even though there are certain advantages to characterizing tumor populations by analyzing cell lines derived from them, we initially chose to characterize the Thy-$1^{lo}$ cells in the primary thymomas in order to minimize phenotypic changes that often occur during adaptation to in vitro culture.

### RESULTS

### Coexpression of B220 and Thy-$1^{lo}$ on immature lymphocytes in primary thymomas induced by A-MuLV

We previously reported that thymomas induced by either v-*abl* or *BCR-ABL* oncogenes usually contain distinct populations of Thy-$1^{hi}$ and Thy-$1^{lo}$ cells (Clark et al., 1993). Further examination revealed that $B220^+$ cells also were present at approximately the same frequency as the Thy-$1^{lo}$ cells (Clark et al., 1993). Therefore, we used two-color flow cytometry to determine whether cells in A-MuLV-induced thymomas coexpress Thy-$1^{lo}$ and $B220^+$. This analysis revealed the presence of two major thymoma subpopulations that could be distinguished by their phenotype. One subpopulation expressed high levels of Thy-1 and was $B220^-$. The second subpopulation was $B220^+$ and expressed Thy-1 at about a 10-fold lower peak fluorescence than the Thy-$1^{hi}$ population (Fig. 1). Thus, the Thy-$1^{lo}$ subpopulation that we previously identified in primary thymomas, also coexpresses B220. However, because the Thy-$1^{lo}$ signal overlaps the isotype controls (data not shown), it is possible that some $B220^+$ thymoma cells may be Thy-$1^-$. In contrast to the thymic $B220^+$ tumor cells, cells from peripheral lymphomas that often appear in thymoma-bearing mice did not express any Thy-1 signal above the isotype background (data not shown) which is typical for A-MuLV-transformed pre-B cells.

To further characterize the $B220^+$ subpopulation, thymoma cells were first enriched over a magnetic column to ≥95% $B220^+$ cells, then examined by immunofluorescent staining for surface Ig, CD2, CD5, CD43 and heat stable antigen (HSA or j11D). The $B220^+$ cells from all thymomas examined expressed a homogenous undifferentiated phenotype (Fig 1 and Table I). Thus, flow cytometry revealed that $B220^+$ thymoma cells did not express surface IgM or Igκ. In addition, Western blot analysis and immunostaining of cytospin preparations both failed to demonstrate expression of Cμ in any of the $B220^+$ tumor subpopulations. In contrast, using these methods we did detect Cμ in normal spleen cells as well as in A-MuLV transformed bone marrow controls (data not shown). Flow cytometry further showed that $B220^+$ tumor cells were $CD5^-$ and $CD2^{neg-lo}$ but expressed uniform levels of both CD43 and HSA (Fig. 1 and data not shown). These phenotypic data are summarized in Table I.

### Both Thy-$1^+$ $B220^-$ and Thy-$1^{lo}$ $B220^+$ thymoma populations arise from A-MuLV-infected cells

To determine whether the different thymoma subpopulations arose from A-MuLV-infected cells, we first attempted to use DNA blot hybridization to examine the pattern of proviral integration. Thymoma populations were sorted on the basis of B220 expression over the magnetic column and a small amount of

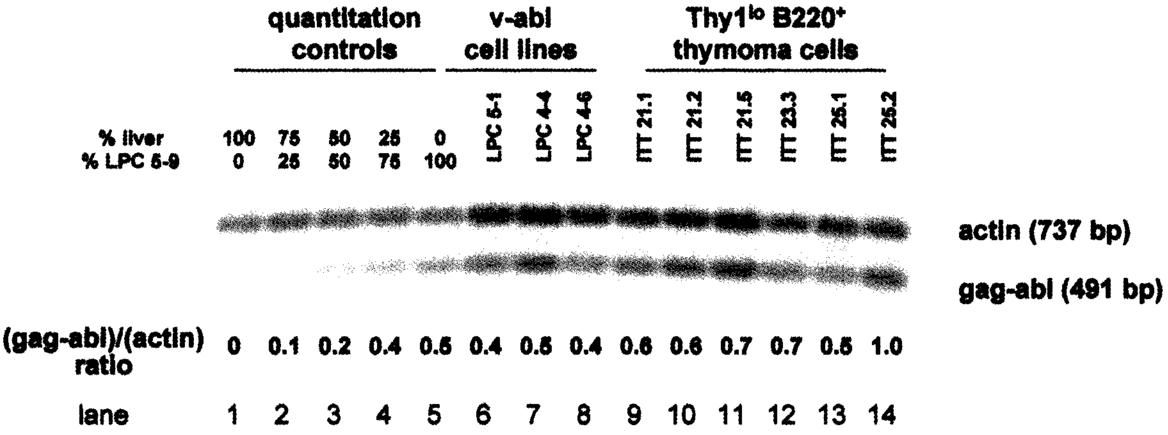

FIGURE 2 Semiquantitative PCR measurement of relative proviral content shows that the B220$^+$ and B220$^-$ thymoma subpopulations both arose from A-MuLV infected cells. After magnetically sorting thymomas into B220$^+$and B220$^-$ fractions, their DNA was amplified using primers that span a 491 bp *gag-abl* fragment and a 737 bp α-actin fragment which served as an internal amplification control. The relative proviral content was determined by normalizing the *gag-abl* signal to the actin signal. Lanes 1–5: Amplification standards were prepared by mixing DNA from normal liver and from the v-*abl* transformed cell line, LPC 5–9, in different proportions. Lanes 5–8: v-*abl* transformed pre-B cell lines. Lanes 9–14: sorted B220$^+$ thymoma cells. Bands were visualized and quantitated by Phosphorimager (Molecular Dynamics) from which the ratio of the *gag-abl* to the actin signal was calculated.

DNA was extracted from the subpopulations. The DNA was digested with *Eco*RI, which does not cut within the A-MuLV genome, and then processed for Southern blot hybridization with a v-*abl* probe. DNA from the B220$^+$ and B220$^-$ subpopulations revealed a 30 kb c-*abl* genomic band along with several unclear bands of submolar hybridization intensity and/or a smear running from about 9–30 kb. These results were repeated on several Southern blots of DNA from column sorted tumor subpopulations (data not shown), which suggests that the subpopulations were oligoclonal with respect to proviral integration, as we previously reported for the unsorted thymomas (Clark et al., 1993). This fact prevented a close comparison of proviral integration in the different subpopulations that coexisted within individual thymomas. On the other hand, DNA obtained from several unsorted peripheral lymphomas revealed single distinct proviral bands for each tumor along with a strong signal from the 30 kb c-*abl* band. Therefore, unlike the thymomas, peripheral lymphomas were clonal in origin.

Because it was difficult to obtain high resolution on DNA blots of the sorted subpopulations, we used the PCR strategy illustrated in Fig. 2 to quantitate the relative proviral content in order to evaluate the contribution of A-MuLV infected cells to each sorted tumor subpopulation. Bands from the PCR reactions were quantitated by Phosphorimager and the relative proviral content of each subpopulation was calculated as the intensity of the 491 bp proviral *gag-abl* band normalized to the intensity of the 737 bp actin band. These values were checked against the proviral contents of standards prepared by serially diluting DNA from a v-*abl* transformed pre-B cell line with DNA from normal liver (Fig. 2, lanes 1–5). In the sorted B220$^+$ (Fig. 3, lanes 9–14) and B220$^-$ (data not shown) tumor subpopulations, the mean relative proviral content was 0.7 (range 0.5–1.0), which was somewhat higher than the 0.5 mean relative proviral content of four clonal v-*abl* transformed bone marrow cell lines (Fig. 3, lanes 5–8, range 0.4–0.5). Nevertheless, this confirmed that both B220$^+$ and B220$^-$ thymoma subpopulations were infected with A-MuLV.

TABLE I

| | Thymoma[a] | Ab staining | | | | | | | | PCR[b] | | DNA blot[c] | | | |
|---|---|---|---|---|---|---|---|---|---|---|---|---|---|---|---|
| | | B220 | Thyl | CD2 | CD5 | CD43 | HSA | sIg | Cμ | $VDJ_H$ | $DJ_H$ | IgH | Igκ | TCRγ | TCRβ |
| A. | 26.9 | pos | lo | lo | neg | pos | pos | neg | | 4% | 24% | | | | |
| | 21.2 | pos | lo | | | | | | | -6% | 29% | | | | |
| | 21.1 | pos | lo | | | | | | | 8% | 44% | | | | |
| | 21.5 | pos | lo | | | | | | | -1% | 48% | | | | |
| | 24.1 | pos | lo | | | | | | | 9% | 58% | | | | |
| | 25.1 | pos | lo | | neg | pos | pos | neg | | 5% | 58% | Rg | GG | GG | GG |
| | 25.7 | pos | lo | lo | neg | pos | pos | neg | neg | 7% | 63% | Rg | GG | GG | GG |
| | 25.9 | pos | lo | neg | neg | pos | pos | neg | | 7% | 66% | Rg | GG | GG | GG |
| B. | 25.2 | pos | lo | | neg | pos | pos | neg | | 23% | 37% | | | | |
| | 26.7 | pos | lo | neg | neg | pos | pos | neg | | 21% | 38% | rG | GG | | |
| | 25.5 | pos | lo | lo | neg | pos | pos | neg | neg | 15% | 60% | Rg | GG | GG | GG |
| C. | 23.9 | pos | lo | | | | | | | 32% | 34% | | | | |
| | 23.3 | pos | lo | | | | | | | 60% | 54% | | | | |
| D. | 26.8 | pos | lo | lo | neg | pos | pos | neg | | | | | | GG | GG |
| | 25.8 | pos | lo | neg | neg | pos | pos | neg | | | | RR | GG | GG | GG |
| | (21.3) | pos | lo | | | | | | neg | | | R(g) | GG | (r)G | GG |
| | (23.5) | pos | lo | | | | | | neg | | | R(g) | GG | (r)G | GG |
| | (24.3) | pos | lo | | | | | | neg | | | R(g) | GG | (r)G | GG |
| | (24.5) | pos | lo | | | | | | | | | R(g) | GG | GG | GG |
| E. | 25.7 | neg | hi | | | | | | | -6% | 15% | GG | GG | Rg | Rg |
| | 24.1 | neg | hi | | | | | | | 2% | 22% | | | | |
| | 25.9 | neg | hi | | | | | | | 2% | 26% | rG | GG | rG | Rg |
| | 25.1 | neg | hi | | | | | | | 5% | 36% | rG | GG | | |
| F. | 25.5 | neg | hi | | | | | | | | | GG | GG | | |
| | 26.8 | neg | hi | | | | | | | | | | | rG | Rg |
| | 26.7 | neg | hi | | | | | | | | | GG | GG | Rg | Rg |
| | (22.7) | neg | hi | | | | | | neg | | | GG | GG | R(g) | R(g) |
| | (22.3) | neg | hi | | | | | | | | | GG | GG | R(g) | R(g) |
| G. | (22.3L) | pos | neg | | | | | | neg | | | RR | GG | GG | GG |
| | (21.3L) | pos | neg | | | | | | neg | | | RR | GG | GG | GG |
| H. | LPC 5–9 | pos | neg | | | | | | pos | 69% | 82% | RR | GG | | |

[a]. Thymoma samples were sorted by anti-B220 magnetic column, except for those marked in parenthesis which were not sorted because they were >85% $B220^+$ or >85% Thy-$1^{hi}$. "L" denotes that the sample was a peripheral lymphoma that arose in an intrathymically injected animal. Thymomas are grouped based upon B220 phenotype and the extent of *Igh* rearrangement as determined by PCR according to the following scheme: (A) $B220^+$ thymomas with only $DJ_H$ rearrangement. (B) $B220^+$ thymomas with less $V_HDJ_H$ than $DJ_H$ rearrangement. (C) $B220^+$ thymomas with equivalent $V_HDJ_H$ and $DJ_H$ rearrangement. (D) $B220^+$ thymomas only analyzed by Southern blot. (E) $B220^-$ thymomas analyzed by PCR. (F) $B220^-$ thymomas only analyzed by Southern blot. (G) $B220^+$ peripheral lymphomas. (H) Bone marrow derived v-*abl* cell line.

[b]. The extent of rearrangement of the *Igh* gene is presented as the mean percent loss of germline structure as illustrated in Fig. 4. The values represent the mean from 3–6 separate amplifications from each sample. Standard errors of the mean percentage loss were <7%. <10% loss of germline is considered as no rearrangement. Rearrangement of the Igκ light chain gene was not detected in any samples tested.

[c]. GG=germline configuration on both alleles, RR=complete rearrangement of both alleles, rG=minor rearrangement detected with predominant germline band, Rg=significant rearrangement detected with minor germline band. Parentheses indicate that the minor band could be attributed to contaminating cells in unsorted thymomas

### Significant subpopulations of Thy-$1^{lo}$ B220$^+$A-MuLV transformed thymoma cells do not demonstrate *Ig* or *TCR* gene rearrangement

In order to assess the extent of antigen receptor gene rearrangement in the Thy-$1^{lo}$ B220$^+$ subpopulation, DNA was extracted from thymoma subpopulations that were sorted for B220 expression on the magnetic column. The DNA was analyzed either by PCR amplification for *Igh* rearrangement or by Southern blot for *TCR* rearrangement. The PCR strategy for *Igh* rearrangement allowed $DJ_H$ and $V_HDJ_H$ rearrangement to be measured as a loss of germline structure relative to unrearranged liver DNA (Fig. 3A). In order to confirm that this analysis was quantitative, liver DNA was serially diluted with DNA from an A-MuLV transformed pre-B cell line, LPC 5.9, amplified by PCR (Fig. 3A), and the loss of *Igh* germline was determined by normalizing the unrearranged *Igh* signal to the amplified actin signal. Figure 3B shows that the measured loss of *Igh* germline is linear and strongly correlates with the expected *Igh* rearrangement (correlation coefficient r = .994 for loss of $V_H$ to $DJ_H$ germline and .997 for loss of D to $J_H$ germline). An example of this analysis is shown in Fig. 3C for sorted subpopulations from thymoma 25.7 (lanes 4 and 5) and from subclones of a marrow derived A-MuLV-transformed cell line that were selected for different patterns of Cμ expression (lanes 2 and 3). The data show that while both B220$^+$and B220$^-$ subpopulations of the thymoma had failed to rearrange $V_H$ to $DJ_H$, the B220$^+$ thymoma subpopulation had undergone significantly more D to $J_H$ rearrangement than the B220$^-$ subpopulation (74% vs 18%). In contrast to the thymoma subpopulations, an A-MuLV transformed pre-B cell line that expressed Cμ (lane 3) showed 74% and 98% loss of $V_H$ to $DJ_H$ and D to $J_H$ segments, respectively, while a less differentiated A-MuLV pre-B cell line that did not express Cμ (lane 2) only lost 22% and 94% of $V_H$ to $DJ_H$ and D to $J_H$ segments, respectively.

Similar PCR analysis showed that 8 of 13 Thy-$1^{lo}$ B220$^+$ subpopulations examined had lost <10% of $V_H$ to $DJ_H$ germline structure (Table IA) which was not statistically different (Student's t-test) from the mean germline content of eight control liver samples. Cells from the eight Thy-$1^{lo}$ B220$^+$ tumors that had not rearranged $V_H$ to $DJ_H$ demonstrated variable $DJ^H$ rearrangement. Two showed 20–30% loss of D to $J^H$ germline structure, two lost 40–50%, and four lost about 60% of the germline structure (Table IA). The remaining five Thy-$1^{lo}$ B220$^+$ thymoma subpopulations that were examined (Table IB and 1C) demonstrated significant $V^H$ to $DJ^H$ rearrangement (15–60%). Three of these subpopulations showed 2–4 fold more rearrangement (37–60%) of D to $J_H$ than $V_H$ to $DJ_H$ sequences (Table IB). In contrast, the Thy-$1^{lo}$ B220$^+$ subpopulation from tumors 23.3 and 23.9 showed approximately equivalent $V_H$ to $DJ_H$ and D to $J_H$ rearrangement (Table IC). Southern blot analysis of *Igh* rearrangement also was performed on some tumor populations, the results of which were in close agreement with the PCR data (Table IA and 1B and data not shown). In addition, four unsorted thymomas that were >85% B220$^+$ were analyzed by Southern blot only which revealed predominantly *Igh* germline structure (Table ID). None of the Thy-$1^{lo}$ B220$^+$ thymoma subpopulations showed rearrangement of *Igκ* light chain genes either by DNA blot or by PCR analyses (Tables IA, 1B, 1C).

The Thy-$1^{lo}$ B220$^+$ thymoma cells also did not demonstrate significant *TCRγ* or *TCRβ* rearrangement by Southern blot analysis (Fig. 4A and B, lanes 1, 3, 5, 7, 8 and data not shown). After hybridizing with the *TCR* probe, the blots were stripped and rehybridized with a c-*myc* probe to normalize for DNA loading (Fig. 4C). Rearrangement of *TCRγ* and *TCRβ* genes was quantitated by measuring the loss of germline bands with a Phosphorimager after normalizing to the level of the c-*myc* gene detected in each lane. Some Thy-$1^{lo}$ B220$^+$ tumor samples showed up to 16% loss of the *TCRγ* or β germline signal (Fig. 4D); however, this minimal rearrangement can be partly attributed to the 5% contamination with Thy-$1^{hi}$ B220$^-$ cells from the magnetic column sorting process. In addition, with this method of quantitation, the level of germline *TCR* DNA detected in seven different liver DNA samples varied by 7% relative to the c-*myc* control (data not shown). Thus, we conclude that in the six Thy-$1^{lo}$

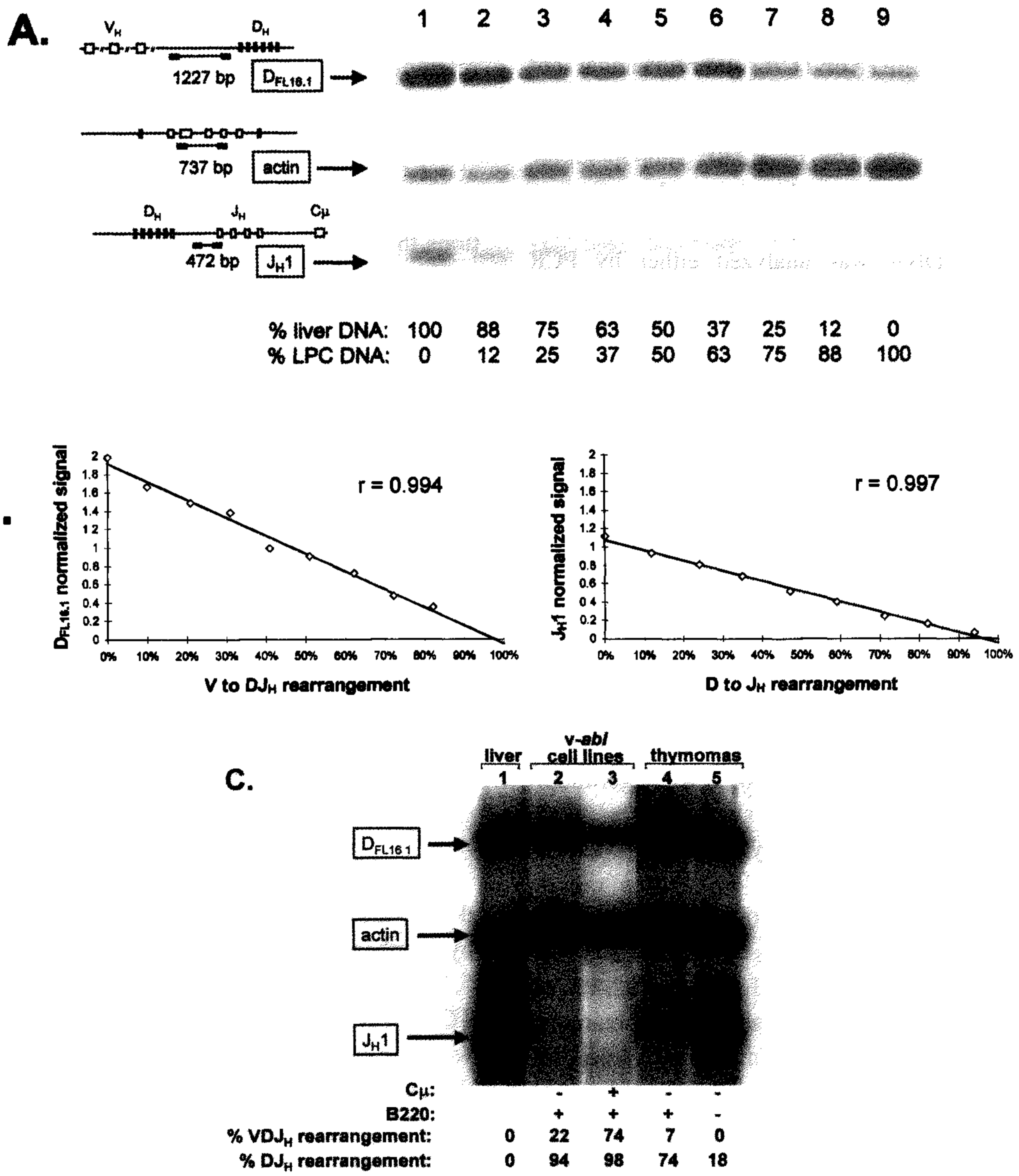

FIGURE 3 (A) Quantitative PCR strategy to measure $V_H$ to $DJ_H$ and D to $J_H$ rearrangement in sorted thymoma populations. The strategy is based upon PCR amplification of intervening regions that are lost upon immunoglobulin assembly so that rearrangement can be measured as the percent loss of germline DNA. Loss of the 472 bp $J_H1$ fragment indicates D to $J_H$ rearrangement and loss of the 1227 bp $D_{FL16.1}$fragment indicates $V_H$ to $DJ_H$ assembly. The 737 bp α-actin fragment was amplified along with immunoglobulin fragments to serve as an internal amplification control for quantitation. DNA from normal liver and a v-*abl* transformed pre-B cell line (LPC 5–1) were mixed together in the indicated proportions and all three sequences were amplified in the same tube. The bands were visualized and quantitated by Phosphorimager. (**B**) The rearrangement of $V_H$ to $DJ_H$ and of D to $J_H$ sequences was measured by normalizing the intensity of each amplified band to the amplified actin product, and this normalized signal was plotted against the expected percent loss of germline structure in the dilution series. The correlation coefficients, r, are indicated and show a linear correlation between the measured loss of germline DNA with the expected degree of *Igh* rearrangement. (C) A representative experiment shows that the magnetically sorted B220$^+$ and B220$^-$ subpopulations from thymoma 25.7 exhibit different degrees of *Igh* rearrangement. Neither subpopulation showed appreciable loss of the $V_H$ to $DJ_H$ germline region, but differed in the degree of rearrangement of D to $J_H$. Lane 1: liver DNA, Lanes 2–3: Cμ$^+$ and Cμ$^-$ marrow-derived A-MuLV pre-B cell lines (both subclones of LPC 5–9). Lanes 4–5: B220$^+$ and B220$^-$ subpopulations sorted from thymoma 25.7. The Cμ and B220 phenotypes, as well as percent loss of $V_H$ to $DJ_H$ and D to $J_H$ germline structure are listed below the figure

$B220^+$ thymoma subpopulations that were examined, negligible rearrangement of *TCR* genes has occurred.

In contrast to the Thy-$1^{lo}$ $B220^+$ thymoma subpopulation, Thy-$1^{hi}$ $B220^-$ tumor cells (Fig. 4A and 4B, lanes 2, 4, 6, and 9) and normal thymus (lane 14) showed extensive rearrangement of both *TCR*γ and *TCR*β genes by DNA blot analysis. *TCR*γ rearrangement was evident by 33–68% loss of germline Jγ bands (Fig 4D, lanes 2, 4, 6, and 9) and by the appearance of rearranged bands (Fig 4A, lanes 2, 4, 6, and 9). *TCR*β rearrangement was also apparent by the 54–59% loss of the Cβ1 germline band (Fig. 4B and 4D, lanes 2, 4, 6, and 9). Since the *TCR*β probe detects the 3' untranslated region of Cβ1 and does not cross-hybridize with Cβ2, complete loss of the Cβ1 germline sequences without the appearance of rearranged bands in these oligoclonal populations is likely due to rearrangement involving Cβ2 (Clark et al., 1993). In contrast to this extensive rearrangement of *TCR* genes in the Thy-$1^{hi}$ $B220^-$ thymoma subpopulation, PCR analysis of *Igh* genes revealed minimal (15–36%) loss of $D_H$ to $J_H$ germline and negligible (<10%) loss of $V_H$ to $DJ_H$ germline sequences, which was confirmed by the DNA blots (Table IE). In the Thy-$1^{hi}$ $B220^-$ thymomà cells, this pattern of minimal *Igh* and extensive *TCR*γ and β rearrangement is consistent with a T-cell lineage origin.

Like the $B220^+$ thymoma subpopulation, the $B220^+$ peripheral lymphomas showed negligible rearrangement of *TCR*γ and *TCR*β (fig 4A and B, lanes 11 and 12). However, unlike the thymoma subpopulation, these $B220^+$ lymphomas were entirely Thy-$1^-$ and demonstrated complete loss of *Igh* germline structure on both alleles by Southern blot (Table IG). This pattern is, as expected, consistent with a B-cell lineage origin of the A-MuLV lymphoma population. None of the tumor populations or cell lines that were examined showed rearrangement of *Ig*κ light chain genes by DNA blot analysis or by PCR analysis (Table I).

### The concentration of A-MuLV can affect the transformation of different thymic targets

The Thy-$1^{lo}$ $B220^+$ and Thy-$1^{hi}$ $B220^-$ tumor subpopulations that co-exist in the thymomas could arise either from the transformation of different thymic lymphocyte targets or by the divergent maturation of a transformed lymphocyte progenitor. We reasoned that if different thymic targets were transformed by A-MuLV, altering the virus titer might affect the selection of different targets for A-MuLV transformation. On the other hand, if both thymoma populations did arise from the same transformed target, then the virus titer would not affect the proportional representation of each population in individual thymomas.

A freshly prepared stock of A-MuLV ($10^5$ ffu) was either concentrated 10-fold or diluted 10-fold in culture media prior to intrathymic injection. The median latent period for tumor formation (35–38 days) was similar in mice injected with the different concentrations of A-MuLV (data not shown). B220 and Thy-1 expression were evaluated by two-color flow cytometry and the data are summarized in Fig. 5. All tumors induced with the diluted (0.1x) virus primarily consisted of Thy-$1^{lo}$ $B220^+$ cells and none of the thymomas in this group were predominately Thy-$1^{hi}$ $B220^-$. In contrast, 70% of the tumors induced with the 10x concentrated virus were almost entirely Thy-$1^{hi}$ $B220^-$, and an intermediate fraction (20%) of tumors from animals injected with the neat virus developed predominately Thy-$1^{hi}$ $B220^-$ thymomas. Although the Thy-$1^{hi}$ $B220^-$ thymomas were phenotypically indistinguishable from thymomas induced by Mo-MuLV alone (Clark et al., 1993), all tumors that developed in mice injected with the different A-MuLV concentrations expressed the $P160^{v\text{-}abl}$ product (data not shown). In addition, all A-MuLV-induced tumors arose after a significantly shorter latent period (range 22–56 days, data not shown) than expected for thymomas induced by intrathymic injection with Mo-MuLV alone (range 81–157 days) (Clark et al., 1993).

### Rare $B220^+$ cells in normal thymi do not contain targets for *in vitro* transformation

In bone marrow a Thy-$1^{lo}$ $B220^+$ B-lymphoid progenitor population is highly enriched for A-MuLV transformation targets (Tidmarsh et al., 1989). Since this phenotype is similar to the A-MuLV-transformed thy-

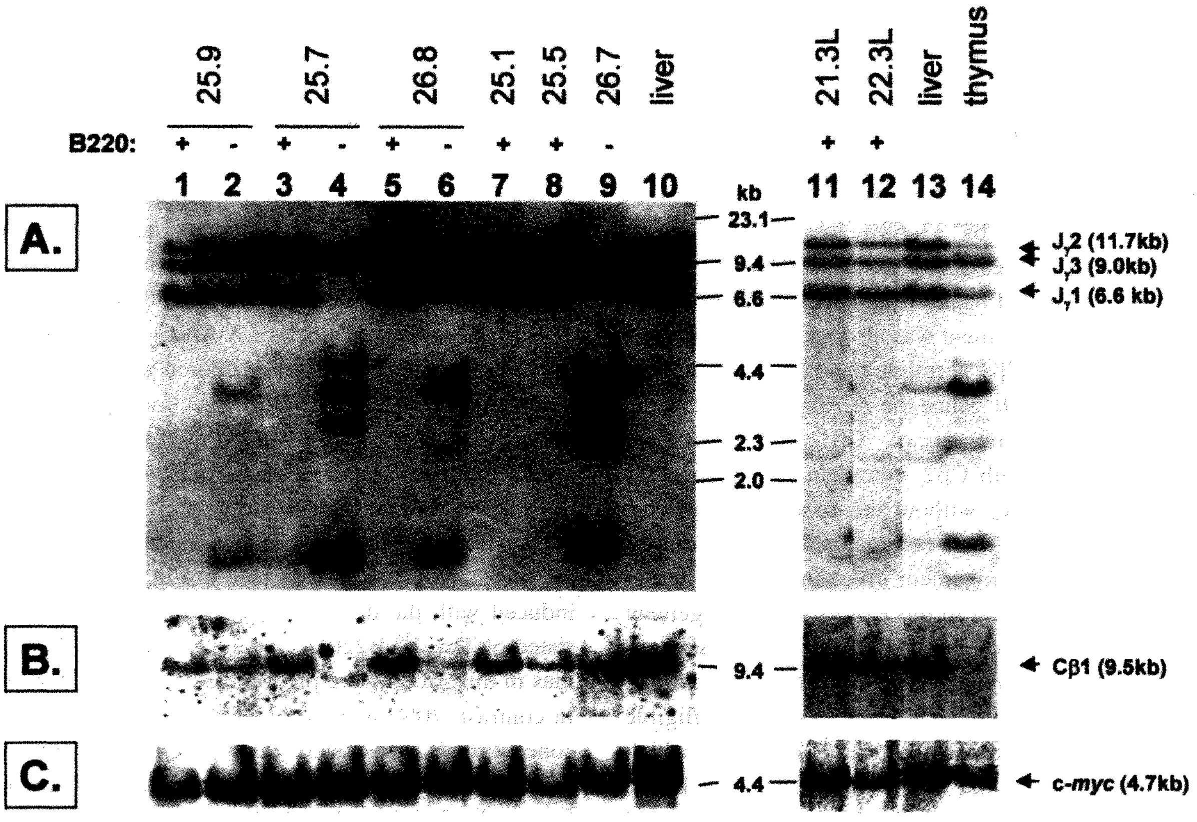

**D.** % loss of germline:

| | 1 | 2 | 3 | 4 | 5 | 6 | 7 | 8 | 9 | 10 | 11 | 12 | 13 | 14 |
|---|---|---|---|---|---|---|---|---|---|---|---|---|---|---|
| *TCRγ* | -7 | 36 | 16 | 68 | 8 | 33 | 4 | -13 | 57 | 0 | -5 | 14 | 0 | 77 |
| *TCRβ* | 13 | 58 | 9 | 54 | -1 | 59 | 5 | -3 | 59 | 0 | 4 | 2 | 0 | 92 |

FIGURE 4 DNA blot analysis of *TCR* rearrangement. *Hin*dIII digested genomic DNA from B220 sorted thymomas (lanes 1–9) or from extrathoracic lymphomas (lanes 11,12) were examined for rearrangement of *TCR*γ (A) and *TCR*β (B). A *c-myc* probe was used to quantitate the relative amount of DNA loaded in each lane (C). In order to measure the loss of germline structure, bands were quantitated by phosphorimaging and the ratio of the *TCR*γ or *TCR*β germline signal to c-*myc* in the tumor populations was compared to a similarly normalized control ratio from unrearranged liver DNA (D). The *TCR*γ germline signal was measured by comparing the signal from individual Jγ1–3 germline bands to the c-*myc* control. Lanes 1 and 2: B220 sorted fractions from thymoma 25.9. Lanes 3 and 4: B220 sorted fractions from thymoma 25.7. Lanes 5 and 6: B220 sorted fractions from thymoma 26.8. Lane 7: $B220^+$ sorted fraction from thymoma 25.1. Lane 8: $B220^+$ sorted fraction from thymoma 25.5. Lane 9: $B220^-$ sorted cells from thymoma 26.7. Lanes 10 and 13: normal liver. Lanes 11 and 12: peripheral lymphomas from mice 21.3 and 22.3, respectively. Lane 14: normal thymus. The arrowheads point to unrearranged germline bands moma population described in this report, we examined normal adult thymi for a similar lymphoid progenitor target susceptible to *in vitro* transformation by A-MuLV. Flow analysis revealed that only 0.3% of all thymocytes express B220. Therefore, the $B220^+$ cells were first enriched approximately 100-fold over an anti-B220 magnetic column and then examined for co-expression of other surface antigens by flow cytometry. This revealed that $B220^+$ thymocytes represent a heterogeneous population in which about 50% express surface IgM and Igκ. About 40% of the thymic $B220^+$ population were also Thy-$1^{lo}$ which resembled the Thy-$1^{lo}$ $B220^+$ thymoma subpopulations as well as the marrow-derived B-cell progenitors that are targets for A-MuLV transformation (Tidmarsh et al., 1989). For this reason, we tested the ability of A-MuLV to transform $B220^+$ thymocytes *in vitro*. As shown in Table II, $B220^+$ bone marrow cells were enriched in targets for *in vitro* transformation by A-MuLV. Conversely, in three different experiments, $B220^+$ cells from normal thymi never showed *in vitro* transformation by A-MuLV, even when plated over a bone marrow stroma with exogenous IL-7, conditions that greatly enhanced transformation of bone marrow pre-B cells (data not shown). In order to test whether contaminating thymocytes in the sorted populations could actively inhibit *in vitro* transformation of a rare pro-B cell, thymocytes and normal bone marrow were admixed prior to infection. No inhibition of *in vitro* transformation was detected (data not shown). Thus, $B220^+$thymic lymphocytes are distinct from $B220^+$ bone marrow lymphocytes in their ability to be transformed *in vitro* by A-MuLV.

### DISCUSSION

### $B220^+$ thymoma cells resemble early lymphoid progenitors

We demonstrated that two distinct tumor subpopulations often co-exist in A-MuLV induced primary thymomas. The subpopulation that was $B220^-$ was also uniformly Thy-$1^{hi}$ and had extensively rearranged *TCR*β, *TCR*γ and $DJ_H$ regions but not $V_H$. This subpopulation, therefore, consists of A-MuLV transformed T-lymphoid cells, similar to A-MuLV-transformed thymic cell lines that have been described by others (Clark et al., 1993; Cook, 1982; Cook, 1985; Cook and Balaton, 1987; Holland et al., 1991; Risser et al., 1985; Scott et al., 1986; Heuze et al., 1992). The other major thymoma subpopulation was $B220^+$ and expressed low levels of Thy-1. This subpopulation had not rearranged *TCR* genes, but had undergone variable rearrangement of *Igh* genes. This population is interesting since it contained a significant proportion of cells that apparently have not rearranged any antigen receptor genes and, therefore, cannot be assigned to either the B or T-lymphoid lineage.

The Thy-$1^{lo}$ $B220^+$ thymoma subpopulation exhibited a composite phenotype that also was consistent with immature lymphoid cells ($CD43^+$ $HSA^+$ $CD5^-$ $CD2^{neg-lo}$ $sIg^-$ $C\mu^-$) but that was not specifically characteristic of either the B or T-lymphoid lineage. Co-expression of Thy-$1^{lo}$ $B220^+$ $CD43^+$ $HSA^+$ and negligible expression of CD2 is consistent with a pro-B cell phenotype (Hardy et al., 1989; Hardy, et al., 1991; Li et al., 1996; Nutt et al., 1997; Rolnick et al., 1996; Sen et al., 1990; Winkler et al., 1995; Yagita et al., 1989) and similar to the phenotype of A-MuLV transformed bone marrow pre-B cells (Rosenberg and Kincade, 1994). However, the fact that a significant proportion of these cells had not rearranged *Igh* genes is inconsistent with the observation that they express HSA which first appears on B lymphocyte progenitors in the process of rearranging the D to $J_H$ region (Allman et al., 1999; Nutt et al., 1997).

TABLE II $B220^+$ thymocytes are not transformed in vitro[a]

| *A-MuLV target population* | *Percentage of total population* | *Number of colonies/$10^6$ plated cells* | *Percentage of total targets*[b] |
|---|---|---|---|
| | | **Bone marrow:** | |
| Unseparated | 100% | 1,028 | 100% |
| $B220^+$ | 40% | 12,273 | 83% |
| $B220^-$ | 60% | 231 | 17% |
| | | **Thymus:** | |
| Unseparated | 100% | 0 | – |
| $B220^+$ | 0.3% | 0 | 0% |
| $B220^-$ | 99.7% | 0 | 0% |

[a] See materials and methods for experimental protocol. Data are representative of three separate experiments.
[b]. The percentage of total targets for a given subpopulation x = $(P_X)/(P_{X1}+ P_{X2} +\ldots + P_{Xn})$ where X1, X2, ..., Xn are n subpopulations defined by flow cytometry and each infected separately with A-MuLV and subsequently plated in soft agar. $P_X$ = (% of subpopulation X in the total unsorted population) × (number of emergent colonies fro subpopulation X, standadized to $10^6$ plated cells)·

The composite phenotype of the Thy-$1^{lo}$ $B220^+$ thymoma subpopulation also is not inconsistent with the phenotype of primitive cortical T-cells. Even though the majority of thymocytes are Thy-$1^{hi}$ $CD2^+$ $CD5^+$ $HSA^{lo}$ (Duplay et al., 1989), an early cortical pro-thymocyte subpopulation expresses a Thy-$1^{lo}$ $CD2^-CD5^-$ $HSA^{hi}$ phenotype (Duplay et al., 1989; Scollay and Shortman, 1985). As the cells mature, expression of CD2 and CD5 increase (Duplay et al., 1989; Sen et al., 1989) while HSA levels decrease (Scollay and Shortman, 1985; Crispe and Bevan, 1987). Additionally, while expression of B220 is more often associated with cells of the B-lymphoid lineage, there are several examples of B220 expression on immature T-cells. For instance, $B220^+$ Thy-$1^{neg-hi}$ pro-T cell lines transformed with v-*abl* (Heuze et al., 1992), v-*myc* (Brightman et al., 1988) and RadLV (Ho and O'Neill, 1993) have been reported. In addition, autoimmune-prone strains of mice often carry $B220^+$ T-lymphocytes (Matsuzaki et al., 1992; Landolfi et al., 1993; Giese et al., 1994) which develop from thymic $B220^+$ progenitor cells (Budd et al., 1985; Asensi et al., 1990). However, while these cells are usually found in autoimmune-prone mice and express CD5, the Thy-$1^{lo}$ $B220^+$ thymoma subpopulation was uniformly $CD5^-$. The B220 antigen was also reported to be expressed on a minor subpopulation of immature $CD4^-8^-$ double negative (DN) thymocytes from normal mice (Gause et al., 1988) as well as on DN thymocytes undergoing apoptosis (Mixter et al., 1999). Finally, rare $B220^+$ lymphoid progenitor cell populations from the bone marrow (Onishi et al., 1993) and from fetal liver (Sagara et al., 1997) reportedly are able to repopulate the thymus (but see Morrison et al., 1994). Therefore, the fact that immature T-cells can sometimes express B220 makes the composite phenotype of the Thy-$1^{lo}$ $B220^+$ thymoma population consistent with immature cells in the T as well as B-lymphoid lineages. We conclude that, based on surface antigen phenotype alone, the A-MuLV transformed Thy-$1^{lo}$ $B220^+$ thymoma cells are not easily assigned to either the B or T-lymphoid lineage.

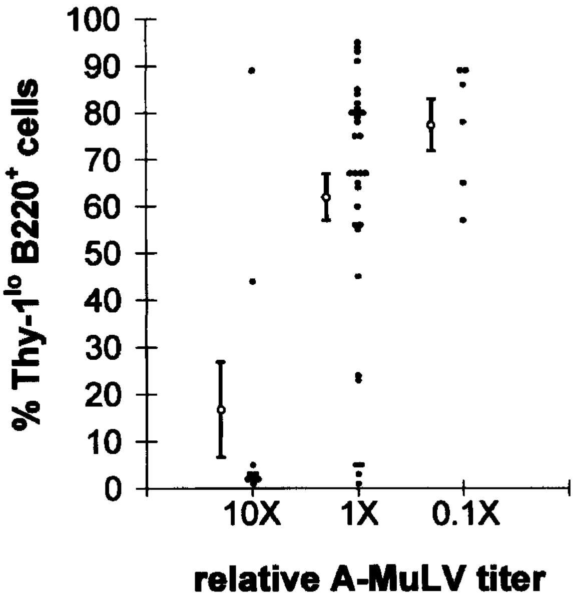

FIGURE 5 The frequency of Thy-$1^{lo}$ $B220^+$ cells in A-MuLV induced tumors correlates with virus titer. Mice were intrathymically injected with the same volume of 10X (n=10), 1X (n=33) or 0.1X (n=6) concentrations of A-MuLV and the percentage of Thy-$1^{lo}$ $B220^+$ cells in each thymoma was determined by flow cytometry. The mean and standard error for the frequency of Thy-$1^{lo}$ $B220^+$ cells in each group is also shown

Overall the immature Thy-$1^{lo}$ B220$^{+}$ cells did not appear to have rearranged TCRγ, TCRβ, or Igκ genes; however, in some thymomas, minor subpopulations of Thy-$1^{lo}$ B220$^{+}$ cells appeared to have undergone $V_H$ to $DJ_H$ rearrangement and, therefore, probably represent transformed thymic pre-B cells similar to those described by others (Cook, 1982; Cook, 1985; Cook and Balaton, 1987; Holland et al., 1991; Kimoto et al., 1989; Risser et al., 1985; Scott et al., 1986). More interesting are those Thy-$1^{lo}$ B220$^{+}$ thymoma populations that, based on the predictable events of *Ig* gene assembly (Schatz et al., 1992), contained a significant number of cells that had not rearranged *Igh* genes. For example, <30% of the Thy-$1^{lo}$ B220$^{+}$ population in thymomas 26.9 and 21.2 showed loss of D to $J_H$ germline structure and no loss of $V_H$ to $DJ_H$ germline sequences (Table IA) and, therefore, appeared to be similar to A-MuLV transformed cell lines that have only rearranged $DJ_H$ (Ramakrishnan and Rosenberg, 1988; Wang and Rosenberg, 1993). This also means that either a maximum of 30% of the population has rearranged both $DJ_H$ alleles or, that up to 60% of the cells have only rearranged one $DJ_H$ allele. Therefore, we conclude that 40–70% of the Thy-$1^{lo}$ B220$^{+}$ cells in these thymomas have not undergone $D_H$ to J rearrangement and may represent transformed undifferentiated lymphocytes.

The Thy-$1^{lo}$ B220$^{+}$ cells in thymomas 23.3 and 23.9 showed a somewhat different pattern of *Igh* rearrangement (Table IC). In both thymomas, these cells apparently lost equal amounts of D to $J_H$ and $V_H$ to $DJ_H$ germline segments. Since $V_H$ rearrangement does not occur until D to $J_H$ assembly has occurred on both alleles (Schatz et al., 1992), it is reasonable to conclude that the cells that showed loss of *Ig* germline structure had completed $V_HDJ_H$ rearrangement on both alleles. It would follow that the remaining cells in these tumors maintain *Igh* genes in germline configuration. For instance, thymoma 23.3 lost about 30% and 40% of D to $J_H$ and $V_H$ to $DJ_H$ germline sequences, respectively. Therefore, the remaining 70% of the cells in the Thy-$1^{lo}$ B220$^{+}$ population in these tumors probably have not undergone *Igh* rearrangement and are undifferentiated lymphocytes.

Thymomas 25.2 and 26.7 showed yet another pattern of *Ig* rearrangement, in which twice as much $D_H$ to $J_H$ germline structure was lost compared to $V_H$ to $DJ_H$ germline structure (Table IB). Both thymomas showed about 40% loss of unrearranged $D_H$ to $J_H$ structure and 20% loss of $V_H$ to $DJ_H$ germline sequences. Since both $DJ_H$ alleles rearrange prior to $V_H$ to $DJ_H$ rearrangement, it is possible that up to 40% of the Thy-$1^{lo}$ B220$^{+}$ thymoma cells have rearranged $D_H$ to $J_H$ on both alleles and $V_H$ to $DJ_H$ on one allele. This would mean that the remaining 60% of the thymoma population have not rearranged *Igh* genes. However, in these tumors, it is also possible that up to 20% of the tumor population completed $V_H$ to $DJ_H$ rearrangement on both alleles. In this case, then, $DJ_H$ rearrangement would be found on another 20–40% of the population, depending on whether the cells rearranged one or both alleles. This means that a minimum of 40% of the cells in these thymoma populations appear to be undifferentiated lymphocytes that have not undergone *Igh* rearrangement.

Together, the quantitative PCR data along with the Southern blot data demonstrate that in some tumors as much as 70% of Thy-$1^{lo}$ B220$^{+}$ cells have not rearranged either *Ig* or *TCR* genes, and, therefore, resemble the earliest lymphoid progenitor. This progenitor-like subpopulation is distinct from most of the previously described A-MuLV-transformed thymoma cell lines, all of which have undergone significant rearrangement of their *Ig* and/or *TCR* genes (Clark et al., 1993; Cook, 1982; Cook, 1985; Cook and Balaton, 1987; Holland et al., 1991; Kimoto et al., 1989; Risser et al., 1985; Scott et al., 1986). Nevertheless, the Thy-$1^{lo}$ B220$^{+}$ progenitor-like cells in the primary thymomas do share similarities with some other transformed lymphoid populations. For instance, these progenitor-like lymphocytes closely resemble thymomas that arise in *bcl-2/myc* transgenic mice (Strasser et al., 1990) and that are Thy-$1^{lo}$ B220$^{+}$ sIg$^{-}$ and have not rearranged T or B lymphocyte antigen receptor genes. It is conceivable that A-MuLV and the *bcl-2/myc* transgenes may transform a similar lymphoid progenitor population. Also, Holland *et al.* (1991), described an A-MuLV-transformed thymoma cell line that was Thy-$1^{neg-lo}$ B220$^{+}$ and that only had minimal *Igh* rearrangement within a subpopulation of the line. These cells were capable of rearranging *TCRγ* genes after intrathymic injection and of continuing *Igh* rearrangement *in vitro*. It is an intriguing possibility that the Thy-$1^{lo}$ $B220^{+}$ progenitor-like cells found in our primary thymomas represent an A-MuLV-transformed thymic lymphocyte progenitor (Carlyle et al., 1997; Wu et al., 1991) that also may be capable of undergoing differentiation along the T and/or B-cell pathways. However, in light of the recent observation that commitment to the B lymphoid lineage may occur before D to $J_H$ recombination (Allman et al., 1999), it is possible that these progenitor-like thymoma cells may already be committed to either the T or B lymphoid lineage. Experiments are underway to attempt to resolve this issue.

### Thymic targets for A-MuLV transformation

The progenitor-like thymoma population expressed a Thy-$1^{lo}$ $B220^{+}$ phenotype that is similar to bone marrow derived B-lymphoid progenitors that are enriched for A-MuLV transformation targets (Tidmarsh et al., 1989). This similarity raised the possibility that a B-cell progenitor in the adult thymus is a target for transformation by v-*abl*. However, unlike the bone marrow $B220^{+}$ cells, thymic $B220^{+}$ cells could not be transformed *in vitro* by A-MuLV. On one hand, it is possible that the thymocyte transformation target that gives rise to the Thy-$1^{lo}$ $B220^{+}$ progenitor-like A-MuLV transformants is not found within the normal $B220^{+}$ thymocyte subpopulation even though 40% of these cells also expressed Thy-$1^{lo}$. However, it is also possible that A-MuLV transformation of a thymic $B220^{+}$ pro-lymphocyte can only occur within a thymic microenvironment, which would explain our inability to transform these cells *in vitro*. Either way, there appear to be fundamental differences between the transformation targets in the bone marrow vs the thymus that give rise to $B220^{+}$ transformed cells.

The co-existence of A-MuLV-transformed pre-B and pre-T cells in primary thymomas raises the question of whether these different populations arise from the transformation of the same or different target cells. The presence of pre-T and pre-B cell transformants in the same thymoma could feasibly arise from A-MuLV infection of separate T and B cell progenitors that may be present in the adult thymus; however, the surprising inability to transform thymic B cell progenitors *in vitro* is not consistent with this explanation. Alternatively, the coexisting pre-T and pre-B cell thymoma populations could feasibly arise from transformation of an undifferentiated lymphocyte progenitor that may undergo T- and B-lymphopoiesis after transformation by A-MuLV (Whitlock et al., 1983). However, it seems unlikely that the coexisting Thy-$1^{hi}$ $B220^{-}$ and Thy-$1^{lo}$ $B220^{+}$ thymoma subpopulations arose from the transformation of a single target cell because intrathymic injection of different concentrations of A-MuLV induced thymomas with distinct phenotypes. If both thymoma populations arose from transformation of a single target cell, it is unlikely that the virus titer would affect their proportional representation in the tumors.

In summary, these observations suggest that a subset of the Thy-$1^{lo}$ $B220^{+}$ thymoma population represents an undifferentiated lymphoid progenitor that cannot be unequivocally assigned to either the T or B-lymphoid lineage. It will be of interest to identify the normal thymocyte target that gives rise to this transformed progenitor population and to assess its capacity for differentiation.

### MATERIALS AND METHODS

### Retrovirus

A-MuLV (1–2 × $10^5$ focus forming units per ml) was collected from clonal NIH 3T3 fibroblast cells infected with a helper-free A-MuLV and superinfected with Moloney murine leukemia virus (Mo-MuLV). To make a concentrated preparation of A-MuLV, viral supernatant was centrifuged over a 20% sucrose cushion. and the virion pellet was gently resuspended in 0.01 volumes of media (Wang et al., 1991). The virus was quantitated by slot blot hybridization which showed a 50-fold enrichment of viral RNA in the concentrated stock; however, quantitation of infectious virus by measuring v-abl oncoprotein expression in acutely infected NIH 3T3 fibroblasts revealed a 10-fold concentration of active virus.

### Tumor cell preparation

Thymic lymphomas were induced in 4–5-week-old female BALB/c mice (Harlan-Sprague-Dawley, Indianapolis, IN) by intrathymic injection of A-MuLV as previously described (Clark et al., 1993). Thymomas arising in individual mice are identified according to the experiment and animal number. Thus, thymoma 25.2 identifies the second animal to develop thymic lymphoma in experiment number 25. Cell suspensions were made from thymomas or peripheral lymphomas and were either processed immediately for immunophenotype analyses or were cryopreserved. Upon thawing cryopreserved samples, dead cells were removed by agglutination in high phosphate buffered saline or by filtration through glass wool (Mishell et al., 1980).

### Preparation of normal thymocytes

3–4 week-old female BALB/c mice were injected intraperitoneally with 0.1 ml India ink diluted 1:5 in PBS. After 20 minutes, mice were sacrificed by $CO_2$ inhalation and perfused by intracardial injection of 10 ml ice cold staining buffer [HBSS (without $CaCO_3$, $MgCl_2$, $MgSO_4$, $NaHCO_3$, or phenol red) containing 0.5% charcoal treated, dialyzed BSA (fraction V, Sigma Chemical Company, St. Louis, MO) and 10 mM $NaN_3$]. The thymus was removed and prepared by removing visible blood vessels and India ink stained parathymic lymph nodes.

### Magnetic cell separation of thymic lymphocytes

For phenotypic and genotypic analyses of $B220^+$ cells in tumor populations that also contained $B220^-$ cells, $B220^+$ cells were separated over a magnetic column as described by others (Miltenyi et al., 1990). Basically, cells were incubated with RA3–6B2-coupled superparamagnetic microbeads (anti-B220; Miltenyi Biotec Inc., Sunnyvale, CA) at 10 μl anti-B220 beads per $10^7$ thymoma cells, or at 10 μl per $10^8$ normal thymocytes. After 10 min., biotinylated RA3–6B2 (Pharmingen, San Diego, CA) (2.5 μg/ml) was added and the cells were incubated for an additional 20 minutes at 4°C. This permitted indirect immunofluorescent detection of B220 expression of column sorted populations. Cells were washed in cold staining buffer and passed over a magnetized A2 separation column according to the manufacturer's protocol (Miltenyi Biotec Inc.). Cells that flowed through were collected as the $B220^-$ fraction. To collect the $B220^+$ fraction, the column was removed from the magnetic field and the adherent cells were washed from the column with cold staining buffer. The efficiency of separation was assessed by flow cytometry after staining with avidin conjugated fluorescein-isothiocyanate (FITC) (Becton-Dickinson, Mountain View, CA). From thymomas with mixed populations of $B220^-$ and $B220^+$ cells, this protocol routinely gave ≥95% $B220^+$ cells from the column and ≤5% in the flow-through fraction. From normal thymi where $B220^+$ cells represented <0.3% of the population, $B220^+$ cells were enriched 100-fold over the column and further purified to homogeneity by FACS.

### Immunofluorescent cell staining and flow cytometry

Immunofluorescent cell staining was performed as described before (Clark et al., 1993), except that cells were preincubated with FcBlock (Pharmingen) in order to prevent potential Fc receptor binding of monoclonal antibodies. Strategies for simultaneous analysis of multiple surface antigens were essentially as described by Hardy *et al.* (Hardy et al., 1991). After cells were flow sorted, an aliquot was reanalyzed by flow cytometry in order to confirm the sorting efficiency. PCR analysis was performed on sorted populations that were 95–98% pure.

### Cell cycle analysis

Simultaneous staining of cell surface antigens and nuclear DNA was done exactly as described previously (Schmid et al., 1991).

### DNA blot analysis

DNA blotting procedures and probes used to analyze tumor clonality and antigen receptor gene rearrangement have been described previously (Clark et al., 1993). The genomic 4.7 kb c-*myc* fragment from pmyc-26 contains c-*myc* exons 1, 2 and 3 and was kindly provided by Dr. Richard Schwartz (Michigan State University).

### PCR quantitation of relative proviral content

In order to measure relative v-*abl* provirus content in different tumor populations, a 491 bp *gag-abl* fragment was amplified by PCR using primers specific for retroviral *gag* (5′ TCC ACT ACC TCG CAG GCA TTC 3′) and *abl* (5′ CCT CCA CCC AAC TTG TGC TTC 3′) sequences. Amplification of a 737 bp region of the α-actin gene (Hardy et al., 1991) was done in the same tube to serve as an internal amplification control. For this PCR analysis, genomic DNA (25 ng) was first digested with *Eco*RI, then treated with 40 μg/ml RNAase (30 minutes at 37°C). The DNA was then added to 100 μl reaction buffer [60 mM Tris-HCl (pH 9.0), 15 mM $(NH_4)_2SO_4$, 1.5mM $MgCl_2$] containing 1 μM of each primer and 0.5 U Taq polymerase (Promega, Madison, WI). Samples were heated to 80°C before 50 μM dNTPs (Promega) were added. The amplification conditions were 95°C for 1.0 minute, 63°C for 0.5 minute, and 72°C for 1.5 minutes for 20 cycles. Five μl of amplification products were electrophoresed through 2.5% agarose and blotted to Gene Screen Plus (NEN Research Products, Boston, MA). PCR amplification products from a v-*abl* transformed pre-B cell line (LPC 5–1) DNA were gel purified and used as probes. The intensities of the *gag-abl* and α-actin amplified signals were quantitated by Phosphorimager (Molecular Dynamics, Sunnyvale, CA).

### PCR analysis of *Ig* rearrangement

In order to measure the extent of *Ig* gene assembly in different tumor populations, quantitative PCR using primers described by Hardy et al. (Hardy et al., 1991) was used to amplify *Ig* gene regions that would be deleted at different stages of rearrangement. $D_H$ to $J_H$ rearrangement was measured by loss of a 472 bp germline segment that is immediately 5′ of $J_H1$. $V_H$ to $DJ_H$ rearrangement was measured by loss of a 1227 bp germline segment 5′ of $D_{FL16.1}$. $V_\kappa$ to $J_\kappa$ rearrangement was measured by loss of a 435 bp germline segment immediately 5′ of $J\kappa1$. PCR amplification of a 737 bp region of the α-actin gene was done in the same tube with the *Ig* primers to serve as an internal amplification control as described above.

The parameters for PCR amplification of *Ig* genes were identical to those described above, except that 0.1 mg of *Eco*RI-digested genomic DNA was used as template, the reaction buffer contained 2.0 mM MgC12, and 23 cycles of amplification were performed. Unrearranged *IgH* and *IgL* products and the actin fragment amplified from liver DNA were used as probes. The *Ig* amplification signal from each tumor sample was normalized to the actin signal amplified in the same tube, and this value was compared to a similarly normalized unrearranged *Ig* amplified signal from liver in order to quantitate the loss of *Ig* germline structure. Each sample was amplified and analyzed 3–6 times.

### Soft agar transformation assay

Bone marrow cells and thymocytes were sorted by FACStar (Becton-Dickinson) based upon B220 and Thy-1 expression. Sorted cells were infected with A-MuLV and then suspended in 0.3% agarose in IMDM containing the following: 20% FCS, $5x10^{-5}$ M 2-ME, 8 mg/ml polybrene, 50 U/ml penicillin G, 50 mg/ml streptomycin sulfate, 400 U/ml recombinant murine IL-7 (Life Technologies, Gaithersburg, MD), 50 U/ml recombinant murine IL-3, and 100 U/ml recombinant murine SCF (both from Immunex, Seattle, WA). The cell suspension was plated over a bone marrow derived stromal cell line (Collins and Dorshkind, 1987). Cultures were fed every 5 days and macroscopic colonies counted 10–14 days later.

### Acknowledgements

The authors are grateful to Larry Morrissey and Kathy Schell for advice and assistance with FACS analyses, and to Drs. Paul Sondel and Robert Auerbach for critical reading of this manuscript.

### References

Akashi K., Kondo M., and Weissman I.L. (1998). Role of interleukin-7 in T-cell development from hematopoietic stem cells. Immunological Reviews 165:13–28.

Allman D., Li J., and Hardy R.R. (1999). Commitment to the B lymphoid lineage occurs before $D_H$-$J_H$ recombination. J. Exp. Med. 189:735–740.

Alt F.W., Yancopoulos G.D., Blackwell T.K., Wood C., Thomas E., Boss M., Coffman R., Rosenberg N., Tonegawa S., and Baltimore D. (1984). Ordered rearrangements of immunoglobulin heavy chain variable region segments. EMBO J. 3:1209.

Antica M., Wu L., Shortman K., and Scollay R. (1994). Thymic stem cells in mouse bone marrow. Blood 84:111–117.

Asensi V., Himeno K., Kawamura I., Sakumoto M., and Nomoto K. (1990). In vivo treatment with anti B-220 monoclonal antibody affects T and B cell differentiation. Clin. Exp. Immunol. 80:268.

Brightman B.K., Chandy K.G., Spencer R.H., Gupta S., Pattengale P.K., and Fan H. (1988). Characterization of lymphoid tumors induced by a recombinant murine retrovirus carrying the avian v-myc oncogene. Identification of novel (B-lymphoid) tumors in the thymus. J. Immunol. 141:2844.

Budd R.C., MacDonald H.R., Lowenthal J.W., Davignon J.L., Izui S., and Cerottini J.C. (1985). Growth and differentiation in vitro of the accumulating Lyt-$2^-$/L3T$4^-$ subset in lpr mice. J. Immunol. 135:3704.

Carlyle J.R., Michie A.M., Furlonger C., Nakano T., Lenardo M.J., Paige C.J. and Zúñiga-Pflücker J.C. (1997). Identification of a novel developmental stage marking lineage commitment of progenitor thymocytes. J. Exp. Med. 186:173–182.

Chen Y.Y., Wang L.C., Huang M.S., and Rosenberg N. (1994). An active v-abl protein tyrosine kinase blocks immunoglobulin light-chain gene rearrangement. Genes & Dev. 8:688.

Clark S.S., Chen E., Fizzotti M., Witte O.N., and Malkovska V. (1993). BCR-ABL and v-abl oncogenes induce distinct patterns of thymic lymphoma involving different lymphocyte sets. J. Virol. 67:6033.

Collins L.S. and Dorshkind K. (1987). A stromal cell line from myeloid long-term bone marrow cultures can support myelopoiesis and B lymphopoiesis. J. Immunol. 138:1082.

Cook W.D. (1982). Rapid thymomas induced by Abelson murine leukemia virus. Proc. Natl. Acad. Sci. USA 79:2917.

Cook W.D. (1985). Thymocyte subsets transformed by Abelson murine leukemia virus. Mol. Cell. Biol. 5:390.

Cook W.D. and Balaton A.M. (1987). T-cell receptor and immunoglobulin genes are rearranged together in Abelson virus-transformed pre-B and pre-T cells. Mol. Cell. Biol. 7:266.

Crispe I.N. and Bevan M.J. (1987). Expression and functional significance of the J11d marker on mouse thymocytes. J. Immunol. 138:2013.

D'Andrea E., Saggioro D., Fleissner E., and Chieco-Bianchi L. (1987). Abelson murine leukemia virus-induced thymic lymphomas: transformation of a primitive lymphoid precursor. J. Natl. Cancer. Inst. 79:189.

Duplay P., Lancki D., and Allison J.P. (1989). Distribution and ontogeny of CD2 expression by murine T cells. J. Immunol. 142:2998.

Gause W.C., Mountz J.D., and Steinberg A.D. (1988). Characterization and differentiation of $CD4^-$, $CD8^-$ thymocytes sorted with the Ly-24 marker. J. Immunol. 140:1.

Gause W.C., Takashi T., Mountz J.D., Finkelman F.D., and Steinberg A.D. (1988). Activation of CD $4^-$, CD $8^-$ thymocytes with IL 4 vs IL 1 + IL 2. J. Immunol. 141:2240.

Giese T. and Davidson W.F. (1994). Chronic treatment of C3H-lpr/lpr and C3H-gld/gld mice with anti-CD8 monoclonal antibody prevents the accumulation of double negative T cells but not autoantibody production. J. Immunol. 152:2000.

Greaves M.F. (1986). Differentiation-linked leukemogenesis in lymphocytes. Science 234:697.

Hardy R.R., Carmack C.E., Shinton S.A., Kemp J.D., and Hayakawa K. (1991). Resolution and characterization of pro-B and pre-pro-B cell stages in normal mouse bone marrow. J. Exp. Med. 173:1213.

Hardy R.R. and Kemp J.D., and Hayakawa K. (1989). Analysis of lymphoid population normal levels in SCID mice by three color flow cytometry with B220 and S7. Curr. Topics. Microbiol. Immunol. 152:19.

Hattori N., Kawamoto H., and Katsura Y. (1996) Isolation of the most immature population of murine fetal thymocytes that includes progenitors capable of generating T, B, and myeloid cells. J. Exp. Med. 184:1901–1908.

Heuze F., Pardoll D. Diez E., Ezine S., and Jotereau F. (1992). Molecular analysis of a pro-T cell clone transformed by Abelson-murine leukemia virus, displaying progessive gamma T cell receptor gene rearrangement and surface expression. Eur. J. Immunol. 22:2077.

Ho E.S. and O'Neill H.C. (1993). T-cell differentiative capacity of haematopoietic stem cells immortalized in vitro with radiation leukemia virus. Leukemia 7:1281.

Holland G.D., Ito K., Kaehler D.A., Tonegawa S., and Risser R. (1991). Thymic targets for Abelson murine leukemia virus are early $\gamma/\delta$ T lymphocytes. Proc. Natl. Acad. Sci. USA 88:3700.

Kawamoto H., Ohmura K., Fujimoto S., and Katsura Y. (1999) Emergence of T cell progenitors without B cell or myeloid differentiation potential at the earliest stage of hematopoiesis in the murine fetal liver. J. Immunol 162:2725–2731.

Kimoto H., Shirasawa T., Taniguchi M., and Takemori T. (1989). B cell precursors are present in the thymus during early development. Eur. J. Immunol. 19:97.

Klug C.A., Gerety S.J., Shah P.C., Chen Y.Y., Rice N.R., Rosenberg N., and Singh H. (1994). The v-abl tyrosine kinase negatively regulates NF-$\kappa$B/Rel factors and blocks $\kappa$ gene transcription in pre-B lymphocytes. Genes & Dev. 8:678.

Kondo M., Weissman I.L., and Akashi K. (1997) Identification of clonogenic common lymphoid progenitors in mouse bone marrow. Cell 91:661–672.

Landolfi M.M., Van Houten N., Russel J.Q., Scollay R., Parnes J.R., and Budd R.C. (1993). $CD2^-CD4^-CD8^-$ lymph node T lymphocytes in MRL lpr/lpr mice are derived from a $CD2^+CD4^+CD8^+$thymic precursor. J. Immunol. 151:1086.

Li Y.-S., Wasserman R., Hayakawa K., and Hardy R.R. (1996) Identification of the earliest B lineage stage in mouse bone marrow. Immunity 5:527–535.

Lian Z., Toki J., Yu C., Hayashi H., Yasumizu R., Sugiura K., Jin T., Inaba M., Hisha H., Li Y., Yu W., Fan H., and Ikehara S. (1997) Intrathymically injected hemopoietic stem cells can differentiate into all lineage cells in the thymus: differences between c-kit$^+$ cells and c-kit$^{<low}$ cells. Stem Cells 15:430–436.

Matsuzaki Y., Gyotoku J., Ogawa M., Nishikawa S., Katsura Y., Gachelin G., and Nakauchi H. (1993). Characterization of c-kit positive intrathymic stem cells that are restricted to lymphoid differentiation. J. Exp. Med. 178:1283.

Matsuzaki Y., Pannetien C., Kanagawa O., Gachelin G., and Nakauchi H. (1992). Evidence for the existence of two parallel differentiation pathways in the thymus of MRL lpr/lpr mice. J. Immunol. 149:1069.

McKenna H.J. and Morrissey P.J. (1998) Flt3 ligand plus IL-7 supports the expansion of murine thymic B cell progenitors that can mature intrathymically. J. Immunol. 160:4801–4809.

Miltenyi S., Müller W., Weichel W., and Radbruch A. (1990). High gradient magnetic cell separation with MACS. Cytometry 11:231.

Mishell B.B., Shiigi S.M., Henry C., Chan E.L., North J., Gallily R., Slomich M., Miller K., Marbrook J., Parks D., and Good A.H. (1980). Preparation of mouse cell suspensions. In Selected methods in cellular immunology, Mishell B.B. and Shiigi S.M., Eds. (New York: W.H. Freeman and Company), p. 3.

Mixter P.F., Russell J.Q., Morrissette, G.J. Charland C., Aleman-Hoey D., and Budd R.C. (1999). A model for the origin of TCR-$\alpha\beta^+$ $CD4^-CD8^-B220^+$ cells based on high affinity TCR signals. J. of Immunol. 162:5747–5756.

Müller-Sieburg C.E., Whitlock C.A., and Weissman I.L. (1986). Isolation of two early B lymphocyte progenitors from mouse marrow: a committed pre-pre-B cell and a clonogenic Thy-1$^{lo}$ hematopoietic stem cell. Cell 44:653.

Nutt S.L., Urbánek P., Rolink A., and Busslinger M. (1997). Essential functions of Pax5 (BSAP) in pro-B cell development: difference between fetal and adult B lymphopoiesis and reduced V-to-DJ recombination at the IgH locus. Genes & Develop. 11:476–491.

Onishi M., Nagayoshi K., Kitamura K., Hirai H., Takaku F., and Nakauchi H. (1993). CD4$^{dull+}$ hematopoietic progenitor cells in murine bone marrow. Blood 81:3217.

Paietta E. (1992). Ambiguous phenotypes in acute leukemia. In Leukemia and lymphoma, Polliack A., Ed. (Philadelphia:Harwood), p. 31.

Péault B., Khazaal I., and Weissman I.L. (1994) *In vitro* development of B cells and macrophages from early mouse fetal thymocytes. Eur. J. Immunol. 24:781–784.

Ramakrishnan L. and Rosenberg N. (1988). Novel B-cell precursors blocked at the stage of $DJ_H$ recombination. Mol. Cell. Biol. 8:5216.

Risser R., Kaehler D., and Lamph W.W. (1985). Different genes control the susceptibility of mice to Moloney or Abelson murine leukemia viruses. J. Virol. 55:547.

Rolink A., ten Boekel E., Melchers F., Fearon D.T., Krop I., and Anderson J. (1996) A subpopulation of B220$^+$ cells in murine bone marrow does not express CD19 and contains natural killer cell progenitors. J. Exp. Med. 183:187–194.

Rosenberg N. and Kincade P.W. (1994). B-lineage differentiation in normal and transformed cells and the microenvironment that supports it. Current Opinion in Immunol. 6:203–211.

Rosenberg N. and Witte O.N. (1988). The viral and cellular forms of the Abelson (abl) oncogene. Adv. Vir. Res. 35:39.

Sagara S., Sugaya K., Tokoro Y., Tanaka S., Takano H., Kodama H., Nakauchi H., and Takahama Y. (1997) B220 expression by T lymphoid progenitor cells in mouse fetal liver. J. Immunol. 158:666–676.

Schatz D.G., Oettinger M.A., and Schlissel M.S. (1992). V(D)J recombination: molecular biology and regulation. Annu. Rev. Immunol. 10:359.

Schmid I., Uittenbogaart C.H., and Giorgi J.V. (1991). A gentle fixation and permeabilization method for combined cell surface and intracellular staining with improved precision in DNA quantification. Cytometry 12:279.

Scollay R. and Shortman K. (1985). Identification of early stages of T lymphocyte development in the thymus cortex and medulla. J. Immunol. 134:3632.

Scott M.L., Davis M.M., and Feinberg B. (1986). Transformation of T-lymphoid cells by Abelson murine leukemia virus. J. Virol. 59:434.

Sen J., Arceci R.J., Jones W., and Burakoff S.J. (1989). Expression and ontogeny of murine CD2. Eur. J. Immunol. 19:1297.

Sen J., Rosenberg N., and Burakoff S.J. (1990). Expression and ontogeny of CD2 on murine B cells. J. Immunol. 144:2925.

Soloff R.S., Wang T.-G., Dempsey D., Jennings S.R., Wolcott R. M., and Chervenak R. (1997) Interleukin 7 induces TCR gene rearrangement in adult marrow-resident murine precursor T cells. Mol. Immunol. 34:453–462.

Strasser A., Harris A.W., and Bath M.L., and Cory S. (1990). Novel primitive lymphoid tumours induced in transgenic mice by cooperation between myc and bcl-2. Nature (Lond.) 348:331.

Tidmarsh G.F., Heimfeld S., Whitlock C.A., Weissman I.L., and Müller-Sieburg C.E. (1989). Identification of a novel bone marrow-derived B-cell progenitor population that co-expresses B220 and Thy-1 and is highly enriched for Abelson leukemia virus targets. Mol. Cell. Biol. 9:2665.

Wang B.C., Kennan W.S., Yasukawa-Barnes J., Lindstrom M.J., and Gould M.N. (1991). Carcinoma induction following direct in situ transfer of v-Ha-ras into rat mammary epithelial cells using replication-defective retrovirus vectors. Cancer Res. 51:2642.

Wang L.C. and Rosenberg N. (1993). RAG-1 and RAG-2 are not sufficient to direct all phases of immunoglobulin gene rearrangement in pre-B cell lines. Mol. Cell. Biol. 13:3890.

Whitlock C.A., Ziegler S.F., Treiman L.J., Stafford J.I., and Witte O.N. (1983). Differentiation of cloned populations of immature B cells after transformation with Abelson murine leukemia virus. Cell 32:903.

Winkler T.H., Melchers F., and Rolink A.G. (1995) Interleukin-3 and interleukin-7 are alternative growth factors for the same B-cell precursors in the mouse. Blood 85:2045–2051.

Wu L., Antica M., Johnson G.R., Scollay R., and Shortman K. (1991). Developmental potential of earliest precursor cells from the adult mouse thymus. J. Exp. Med. 174:1617.

Yagita H., Nakamura T., Asakawa J.I., Matsuda H., Tansyo S., Iigo Y., and Okumura K. (1989). CD2 expression in murine B cell lineage. Int. Immunol. 1:94.

